# HIV prevalence by ethnic group covaries with prevalence of herpes simplex virus-2 and high-risk sex in Uganda: An ecological study

**DOI:** 10.1371/journal.pone.0195431

**Published:** 2018-04-04

**Authors:** Chris R. Kenyon

**Affiliations:** 1 HIV/STI Unit, Institute of Tropical Medicine, Antwerp, Belgium; 2 Division of Infectious Diseases and HIV Medicine, University of Cape Town, Cape Town, South Africa; David Geffen School of Medicine at UCLA, UNITED STATES

## Abstract

**Background:**

HIV prevalence varies from 1.7% to 14.8% between ethnic groups in Uganda. Understanding the factors responsible for this heterogeneity in HIV spread may guide prevention efforts.

**Methods:**

We evaluated the relationship between HIV prevalence by ethnic group and a range of risk factors as well as the prevalence of herpes simplex virus-2 (HSV-2), syphilis and symptomatic STIs in the 2004/2005 Uganda HIV/AIDS Sero-Behavioural Survey—a two stage, nationally representative, population based survey of 15–59-year-olds. Spearman’s correlation was used to assess the relationship between HIV prevalence and each variable.

**Results:**

There was a positive association between HIV prevalence and HSV-2, symptomatic STIs and high-risk sex (sex with a non-cohabiting, non-marital partner) for women. Non-significant positive associations were present between HIV and high-risk sex for men and lifetime number of partners for men and women.

**Conclusion:**

Variation in sexual behavior may contribute to the variations in HIV, HSV-2 and other STI prevalence by ethnic group in Uganda. Further work is necessary to delineate which combinations of risk factors determine differential STI spread in Uganda.

## Introduction

The importance of population level studies is increasingly being appreciated in STI epidemiology [1–4]. Whereas individual level risk factors are critical in determining who within a population is at risk of STI acquisition, population level attributes (such as the connectivity of the populations sexual network) determine the prevalence of STIs [3, 5]. Sexual network connectivity is in turn determined by a number of factors such as number of sex partners and partner concurrency [5]. Because these are population level attributes, ecological studies are required to assess the association between markers of network connectivity and STI prevalence [4, 5].

Studies from Ethiopia [6], Kenya [7], South Africa [8, 9] and the United States [1],have revealed that certain ethnic groups have higher prevalences of most or all STIs measured. Population level studies from these countries have generally found that the ethnic groups with the higher STI rates have more connected sexual networks [1, 6, 7, 9]. These insights have then led to interventions to address these risk factors [10].

It has long been noted that the spread of HIV in Uganda has been uneven between regions and ethnic groups [11–14]. Numerous studies have established that a range of individual level risk factors such as number of partners and age at sexual debut are risk factors for HIV infection in Uganda [13–19]. We could not, however, find any studies that have attempted to explain the heterogeneity in HIV spread at a population level.

In this paper we therefore make use of a nationally representative sample of Ugandans that measured HIV, herpes simplex virus-2 (HSV-2), syphilis and sexual behavior to answer two ecological-level questions at the level of ethnic groups. Firstly, is there an association between HIV prevalence and sexual behavior? Secondly, is the prevalence of HIV associated with of the prevalence of HSV-2, syphilis and symptomatic STIs?

## Methods

The 2004/2005 Uganda HIV/AIDS Sero-Behavioural Survey (UHSBS) was a two stage, population based survey of 15–59-year-olds. The survey aimed at being nationally representative but its representativeness was not verified. All participants were interviewed about a range of demographic, social and behavioural items and were then asked to voluntarily provide a blood sample for the purpose of testing for HIV, syphilis and herpes simplex virus 2 (HSV-2). 11454 women and 9905 men from 9529 households in 417 enumeration areas were eligible to participate in the survey. A high proportion of these individuals completed the questionnaire (95% of women and 89% of men) and provided blood samples (90% of women and 84% of men).

### Blood sampling/processing and testing

Venous blood was collected in a 4.5 ml EDTA Vacutainer tube. The blood was then transported in a cold box to a temporary field laboratory that was set up in each study area. The blood was then centrifuged and the plasma transferred to microvials. The microvials were stored in liquid nitrogen tanks and transported to the Uganda Virus Research Institute (UVRI) where they were subjected to the following tests:

Herpes simplex-2 (HSV-2): Specimens were tested using an HSV-2 EIA (Kalon Biological HSV Type 2 IgG indirect ELISA). Specimens with results in the ‘grey zone’ were tested again. Specimens that remained in the ‘grey zone’ on retesting were reported as ‘indeterminate’ and reported as negative in this manuscript. The quality control step involved retesting all positive samples and 5 percent of the negatives at the CDC-Uganda lab.

HIV: Specimens were tested with a two HIV EIA parallel testing algorithm—Murex 1.2.0 (Abbott) and Vironostica Uniform II Plus O (Biomerieux). Testing was repeated for specimens with ‘grey zone’ or discordant results with the two assay testing. Western blot was then carried out for samples with repeatedly discordant results. As a quality control step, all positive specimens and 5 percent of all negative specimens were retested by the CDC-Uganda lab using the same testing algorithm.

Syphilis: All plasma specimens were screened with the rapid plasma reagin (RPR) test at a dilution of 1:8. They were reported as positive after review by a second reader. All RPR positive specimens and 10 percent of negative specimens were also tested with the treponemal pallidum haemaglutination assay (TPHA) test. A quality control step involved retesting all positive specimens and 5 percent of negative specimens at the CDC-Uganda lab.

Further details of the survey design are presented elsewhere [13]. The UHSBS received ethical committee clearance for data analyses such as the one performed here. As a result, no specific ethics committee approval was necessary for this study.

Each of the following variables was calculated separately for men and women and were limited to those between the ages of 15 and 49 years. The age range 15–49 was selected as this the most commonly used age range in similar studies [7, 9, 20]. The two exceptions were men circumcised which was limited to men and HIV prevalence which reflects the combined HIV prevalence of men and women 15–49 years old.

High-risk sex: Percentage of women/men who have had sex with a non-marital, non-cohabiting partner in the last 12 months of all 15–49 year old women/men reporting sexual activity in the last 12 months
Multiple partners in past-year: Percentage of women/men aged 15–49 who have had sexual intercourse with more than one partner in the last 12 months.
Lifetime partners: Mean number of sexual partners in her lifetime among 15–49 year old women/men who ever had sexual intercourse.
Men circumcised: Percentage of 15–49 year old men who report being circumcised.
Symptomatic with STIs: Percentage of women/men aged 15–49 who report either a genital ulcer or a vaginal/urethral discharge in the last 12 months.
HIV prevalence: Percentage of 15–49 year old men and women who tested positive for HIV.
HSV-2 prevalence: Percentage of 15–49 year old men and women who tested positive for HSV-2.
Syphilis prevalence: Percentage of 15–49 year old men and women who tested positive for syphilis (Screening with Rapid Plasma Reagin >1/4 and confirmation of positive results with Treponema pallidum Hemagglutination Assay).

Homophilous partnering by ethnic group: The available datasets only collected information about the ethnicity of live–in-husbands. As a result, the degree of ethnic-homophily per ethnic group was defined as the percentage of married women whose husbands were from the same ethnic group. Ethnicity was measured by self-reporting.

### Statistical analysis

All analyses are ecological in nature and conducted by ethnic group separately for men and women. The analyses were conducted using STATA 13.0 (College Station, TX). All prevalence estimates have been adjusted to account for the complex sampling strategies of the survey using the survey (SVY) command. Associations were assessed using Spearman’s correlation.

## Results

The prevalence of HIV varied from 1.7% (95% CI 1.0–2.8) in the Karimojong to 14.8% (95% CI 10.7–19.9) in the Batoro (Table 1). HSV-2 prevalence varied from a low of 28.7%/31.2% in the Lugbara/Karimojong to a high of 54.4% in the Batoro. The prevalence of syphilis was highest in the Karimojong (8.6%) and lowest in the Lugbara/Madi (1.9%). Homophily rates by ethnic groups were high (median 81.1%, IQR 78–94%; Table 2). Most ethnic groups were concentrated in one or two contiguous regions (Table 3). There was little variation in the age structure of the ethnic groups (Table 4).

**Table 1:** HIV prevalence by ethnic group and gender (2004/2005 Uganda HIV/AIDS Sero-Behavioural Survey).

	Women 15–49		Men 15–49		Total	
	HIV positive(%)	N Tested	HIV positive(%)	N Tested	HIV positive(%)	N Tested
**Baganda**	10.1	1672	5.8	1304	8.2	2976
**Banyankore**	7.6	966	5.9	776	6.9	1742
**Iteso**	5.1	607	4.7	495	4.9	1101
**Lugbara/Madi**	3.2	742	2.2	562	2.8	1304
**Basoga**	5.6	893	5.6	685	5.6	1577
**Langi**	11.3	478	7.3	432	9.4	910
**Bakiga**	8.5	634	4.1	538	6.5	1172
**Karimojong**	2.1	284	1.1	188	1.7	472
**Acholi**	7.1	468	6.7	343	6.9	810
**Bagisu/Sabiny**	7.5	426	3.5	450	5.4	876
**Alur/Jopadhola**	8.0	484	4.3	414	6.3	899
**Banyara**	7.4	304	6.8	247	7.1	551
**Batoro**	16.4	230	12.8	198	14.8	428
**Others**	6.5	1156	3.2	835	5.1	1992
**Total**	7.5	9391	5.0	7515	6.4	16906

**Table 2:** Homophily by ethnicity. The self-defined ethnicity of married husbands and wives in the 2004/2005 Uganda HIV/AIDS Sero-Behavioural Survey.

	Husband’s Ethnicity															
Wife’s Ethnicity	Baganda	Banyankore	Iteso	Lugbara/Madi	Basoga	Langi	Bakiga	Karimojong	Acholi	Bagisu/Sabiny	Alur/Jopadhola	Banyara	Batoro	Others	Total	Percent Homophily
Baganda	373	10	4	2	23	0	7	0	0	5	5	9	7	33	478	78.0
Banyankore	22	323	1	1	1	0	43	0	0	0	2	2	3	11	409	78.9
Iteso	3	0	439	0	9	3	0	1	0	2	3	0	0	7	467	94.0
Lugbara/Madi	2	0	2	502	2	0	1	0	2	0	8	1	0	9	529	94.8
Basoga	17	0	14	1	341	0	3	0	0	5	9	2	0	22	414	82.3
Langi	3	0	1	1	0	338	0	0	4	0	2	1	0	7	357	94.6
Bakiga	7	32	0	0	0	0	253	0	0	1	1	3	7	15	319	79.3
Karimojong	0	0	0	0	0	0	0	322	0	0	0	0	0	0	322	100.0
Acholi	1	0	1	0	0	7	0	0	273	0	2	0	1	2	287	95.1
Bagisu/Sabiny	5	0	4	0	11	1	0	0	0	259	1	0	0	6	287	90.2
Alur/Jopadhola	4	0	17	7	7	2	0	0	2	0	249	11	1	12	312	79.8
Banyara	14	0	3	4	2	0	1	0	0	1	1	96	8	6	136	70.5
Batoro	3	6	0	1	1	1	4	0	0	0	1	3	74	5	99	74.7
Others	40	16	19	18	37	6	15	0	0	16	19	7	11	367	571	64.2
Total	494	387	505	537	434	358	327	323	281	289	303	135	112	502	4,987	

**Table 3:** Distribution of ethnic groups in regions in 2004/2005 Uganda HIV/AIDS Sero-Behavioural Survey.

	Region									
Ethnicity	Central	Kampala	East Central	Eastern	Northeast	North Central	West Nile	Western	Southwest	Total
Baganda	1143	1085	450	15	0	3	3	21	55	2775
Banyankore	218	184	15	1	0	0	0	176	1070	1664
Iteso	24	40	88	189	1236	6	2	0	0	1585
Lugbara/Madi	21	52	32	0	0	12	2135	39	3	2294
Basoga	31	101	1124	319	7	2	0	3	2	1589
Langi	1	18	5	2	2	1168	1	7	0	1204
Bakiga	70	73	8	0	0	0	1	366	686	1204
Karimojong	0	0	0	0	1006	0	1	0	0	1007
Acholi	4	43	7	2	0	871	4	61	0	992
Bagisu/Sabiny	4	42	71	901	4	2	1	4	0	1029
Alur/Jopadhola	23	68	126	346	1	11	468	90	0	1133
Banyara	59	53	19	2	1	5	2	418	1	560
Batoro	9	71	6	1	0	1	3	410	0	501
All others	341	160	273	175	116	40	178	545	269	2097
Total	1948	1990	2224	1953	2373	2121	2799	2140	2086	19634

**Table 4:** Age structure by ethnic group and gender in 2004/2005 Uganda HIV/AIDS Sero-Behavioural Survey.

	Men			Women		
	15–29 years N (row %)	30–49 years N	Total	15–29 years N (row %)	30–49 years N	Total
Baganda	713 (64.2)	397	1110	994 (67.3)	484	1478
Banyankore	405 (59.3)	278	683	510 (60.3)	336	846
Iteso	351 (53.8)	301	652	454 (57.6)	334	788
Lugbara/Madi	569 (60.6)	370	939	723 (60.7)	468	1191
Basoga	329 (52.1)	302	631	488 (58.9)	341	829
Langi	270 (52.6)	243	513	306 (54.3)	258	564
Bakiga	290 (58.5)	206	496	341 (58.1)	246	587
Karimojong	152 (41.2)	217	369	293 (53.5)	255	548
Acholi	197 (50.9)	190	387	273 (53.7)	235	508
Bagisu/Sabiny	251 (53.7)	216	467	245 (54.7)	203	448
Alur/jopadhola	281 (57.8)	205	486	334 (59.6)	226	560
Banyara	142 (59.2)	98	240	175 (62.7)	104	279
Batoro	123 (55.7)	98	221	149 (60.6)	97	246
Ohers	449 (56.1)	352	801	641 (58.6)	453	1094

### Associations between STIs

**Fig 1:**
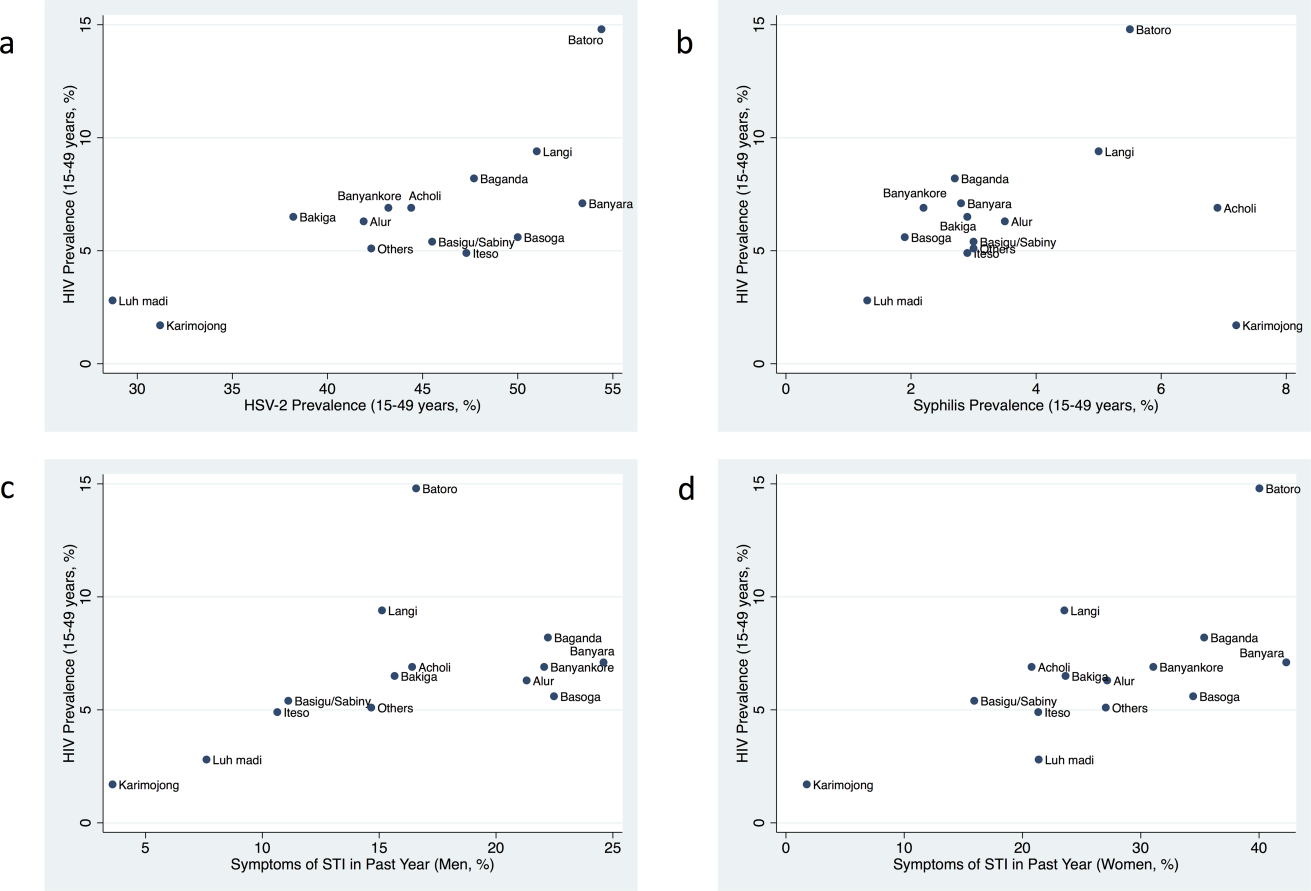
Association between adult HIV prevalence (15–49 years) and HSV-2 prevalence (a) syphilis prevalence (b), percent with STI symptoms in past year in men (c) and women (d) in the 2004/2005 Uganda HIV/AIDS Sero-Behavioural Survey. All data are by ethnic group and limited to those 15–49 years old.

The prevalence of HIV was positively associated with HSV-2 (rho = 0.70, P = 0.005) and symptomatic STIs reported in women (rho = 0.63, P = 0.015) and men (rho = 0.67, P = 0.008, Fig 1). HIV was not associated with syphilis prevalence (Fig 1).

### Associations between HIV prevalence and risk factors

**Fig 2:**
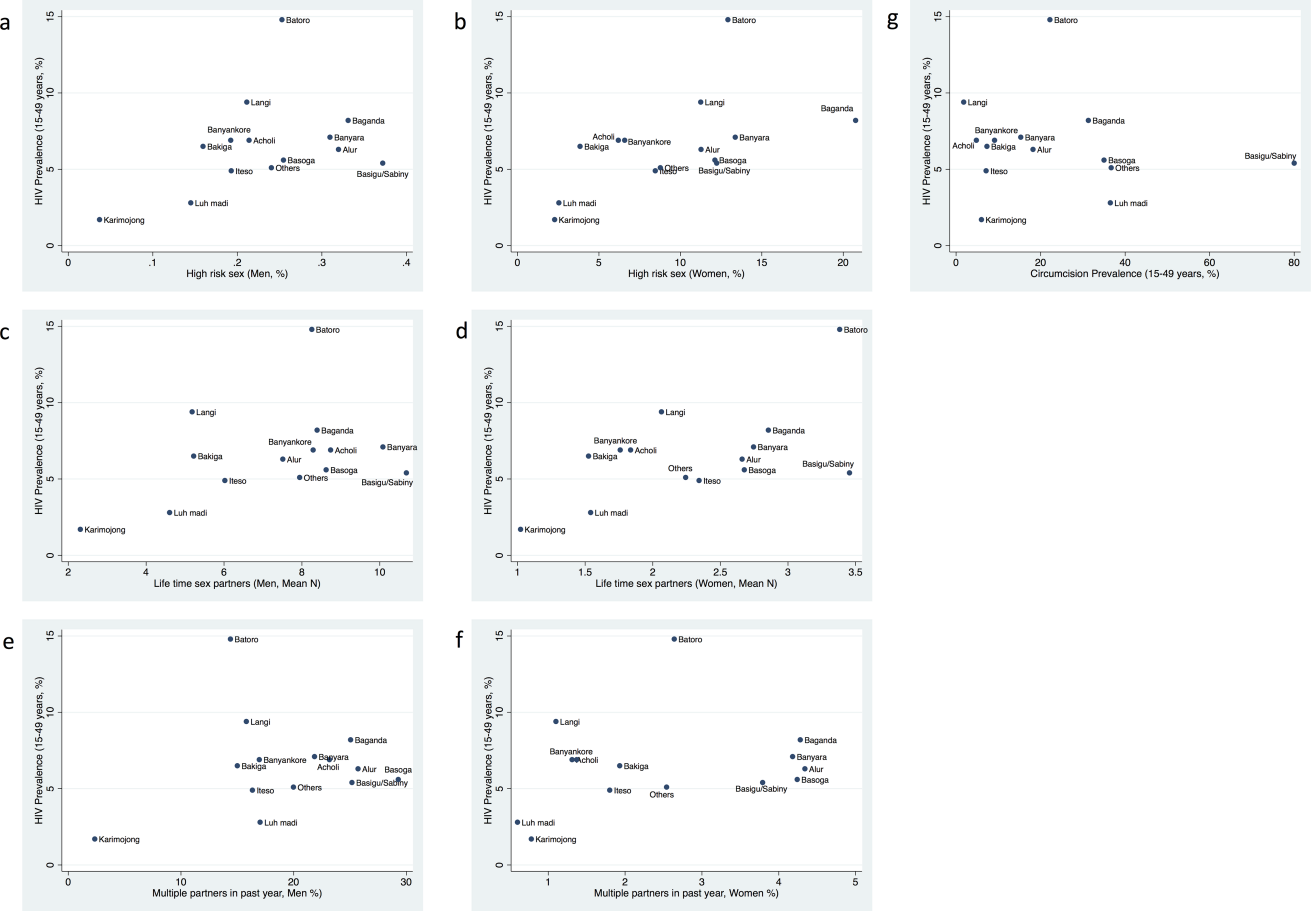
Association between adult HIV prevalence (15–49 years) and high-risk sex in men (a) and women (b), mean number of lifetime partners for men (c) and women (d), percent reporting multiple partners in past year for men (e) and women (f) and circumcision prevalence (g) in the 2004/2005 Uganda HIV/AIDS Sero-Behavioural Survey. All data are by ethnic group and limited to those 15–49 years old.

A positive association was found between the prevalence of HIV and high-risk sex for women (rho = 0.59, P = 0.027, Fig 2, Table 5). Non-significant positive associations were present between HIV and high-risk sex for men, multiple partners in past year for women and lifetime number of partners for men and women. The prevalence of male circumcision was not associated with HIV prevalence (rho = -0.23, P = 0.427).

**Table 5:** The association between HIV prevalence in 2004/2005 Uganda HIV/AIDS Sero-Behavioural Survey and various risk factors by ethnic group.

	Men ^a^	Women ^a^
High-risk sex	0.39	0.59*
Multiple partners in past-year	-0.02	0.31
Lifetime partners	0.37	0.40
Men circumcised	-0.23	NA

^a^ Spearman's correlation was used for these comparisons.

* P<0.05

## Discussion

We found positive associations between HIV, HSV-2 and symptomatic STIs but not syphilis. There are a number of possible explanations for this discrepancy. Although most previous individual and ecological studies have found positive associations between HIV and syphilis [20–24], a number of studies have found that in populations heavily affected by AIDS mortality this association may be lost or even reversed [25–27]. This is thought to be due to AIDS mortalities effect on disrupting sexual network connectivity and hence syphilis incidence [25]. Because HSV-2 serological tests remain positive lifelong this network-disruption effect is less likely to impact on HSV-2 prevalence [28].

**Fig 3:**
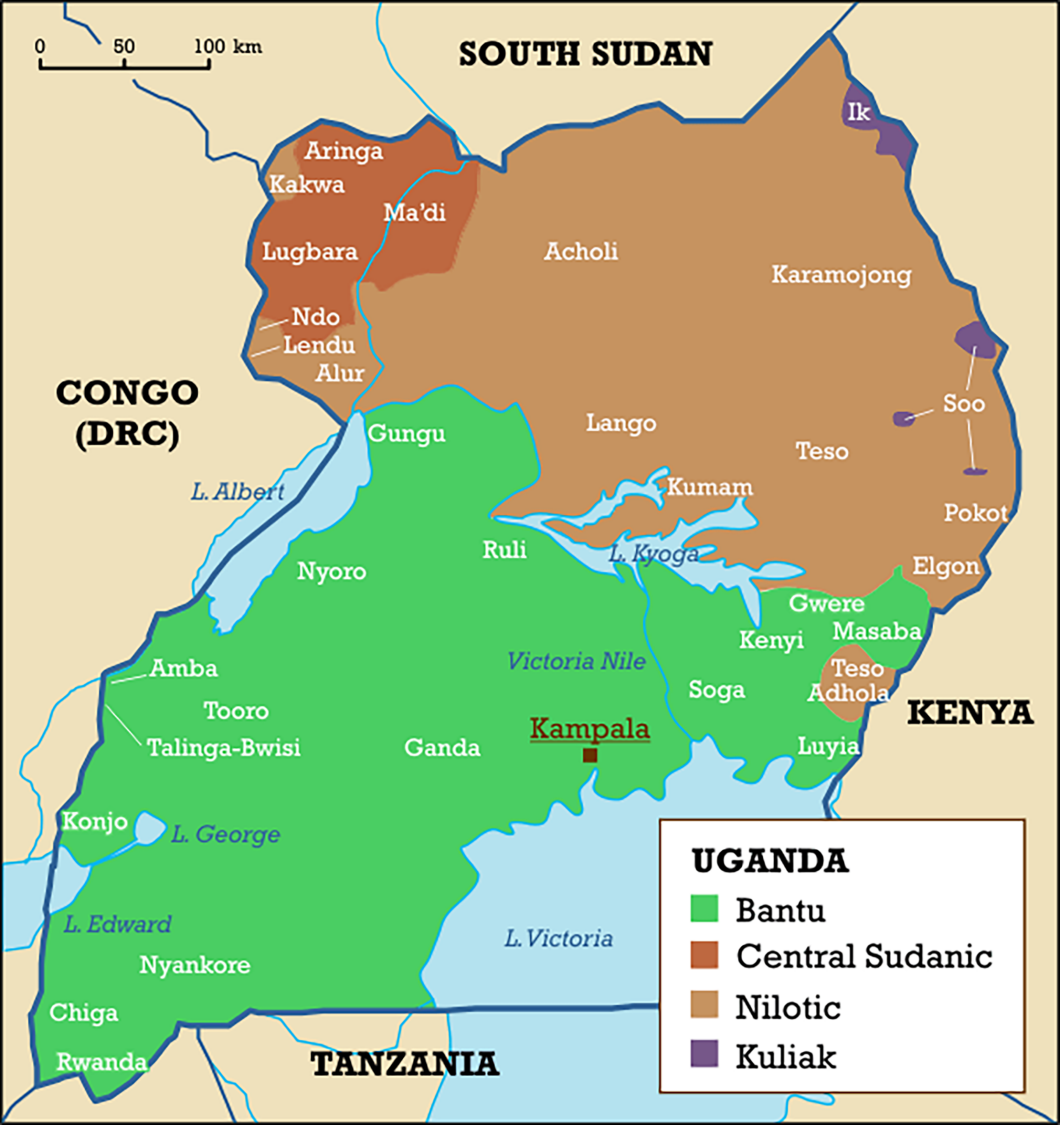
Map of Uganda showing the distribution of ethnolinguistic groups. Reprinted from [46] under a CC BY license, with permission from Mark Dingemanse, original copyright 2005.

Alternatively yaws, which is indistinguishable from syphilis using serological testing, may be responsible for the high ‘syphilis’ prevalence in the two outlying groups with high ‘syphilis’ but lower HIV prevalence (Karimojong and Acholi). These two groups live in contiguous regions in the north of Uganda where yaws has long been endemic [29, 30]. One of the earliest papers describing this compared the case rates of syphilis and yaws (both diagnosed clinically) presenting to all clinics in the Lango District (North Central region of Uganda and home to the Acholi, Fig 3) with Masaka (Central Region inhabited mainly the Buganda) between 1928 and 1938 [30]. Striking differences were noted. The incidence of yaws in Lango was 13 times that at Masaka, whereas the incidence of syphilis was 16 times higher in Masaka. The low rates of high-risk behavior, HSV-2 prevalence and symptomatic STIs in the Karimojong are all compatible with this explanation. According to this interpretation, the marked difference in syphilis incidence between Masaka and Lango in the 1930s may have been a marker of risk for subsequent HIV and other STI epidemics as has been found for other populations in Africa and the United States [31]. Differential syphilis control or usage of antibiotics for other indications are other possible explanations.

Our analysis is subject to a number of limitations. It is an ecological analysis and thus susceptible to the ecological inference fallacy. The UHSBS was designed to provide accurate estimates of national and regional but not ethnic group level prevalence. We did not validate how representative the UHSBS sample was at these levels. Our assessment of the factors underpinning divergences in HIV prevalence were crude and subject to a range of misclassification biases. In particular the composition of sexual networks is determined by a range of factors [32]. We have only evaluated differences in STIs and behaviours by ethnic group. This is defensible in Uganda, because rates of homophily by ethnic group are high. Nonetheless even in places like this, multiple other factors influence the connectivity of individual networks and the connectivity between networks [32, 33]. We were only able to evaluate a very limited number of markers of sexual behavior risk. A further important misclassification bias is related to the inaccuracies of the STI testing algorithms used. In particular, HSV-2 serological testing including the testing algorithm used in the UHSBS is characterized by relatively poor sensitivity and specificity [34, 35]. A metanalysis of the performance of HSV-2 serological testing in sub Saharan Africa found that the Kalon HSV-2 EIA used in the UHSBS had a sensitivity of 95% (95% CI: 93%–97%) and a specificity of 91% (95% CI: 86%–95%) [35]. Furthermore, this paper has not explored the upstream determinants of sexual behavior. Ethnic groups were not all equally affected to the same extent by various disruptions over the past few decades such as the Ugandan Civil War, the activities of the Lord’s Resistance Army and the Ugandan government's response to these.

Other surveys have found a similar distribution of HIV by region/ethnic group in Uganda [14, 17, 18, 36]. One cross sectional survey in the Fort Portal municipality (which is mainly inhabited by the Batoro) found that number of sex partners and being a member of the Batoro were independently associated with HIV status [17]. We found that ethnic groups with higher HIV prevalences tended to report higher number of partners and be more likely to have sex with persons that were not living with them or married to them. Both these risk factors have been found to be associated with HIV at an individual level in the 2004/5 UHSBS, the 2011 Uganda AIDS Indicator Survey [13, 14] and most other sub Saharan African countries where this has been assessed [37]. Our findings that these risk factors correlate with HIV prevalence at population level are similar to those from studies in Ethiopia, Kenya, South Africa the USA [6, 7, 9, 22, 23]. Our results are also commensurate with those of a study that found a borderline positive association between lifetime number of partners and HIV prevalence by region in Uganda in 2011 [38]. Taken together, these findings suggest that differences in sexual behavior offer a parsimonious explanation for the large differences in HIV prevalence between regions and ethnic groups in Uganda. Considerable further study is however required to unpack exactly which aspects of behavior are most responsible.

Apart from the National Health and Nutrition Examination Surveys (NHANES) from the USA and the UHSBS we know of no other nationally representative samples that measure HSV-2 serology as well as HIV. Evaluations of the NHANES surveys and a local survey in Masaka, Uganda, have found a strong association between HIV and HSV-2 prevalence by ethnic group [18, 23, 39]. South Africa has recently included HSV-2 serology instead of syphilis serology in its antenatal surveys [40]. This confirmed the finding of earlier studies that found a strong correlation between HIV and HSV-2 serology by ethnic/racial group [23, 40, 41]. A number of studies have found that populations with higher HSV-2 prevalence have more connected sexual networks (as reflected by concurrency prevalence and number of sex partners) [23, 39, 41, 42]. One analysis for example, found that populations with HSV-2 prevalence below 20% in 20 to 24 year women had an extremely low probability of developing generalized HIV epidemics [41]. The close association between HSV-2 and HIV in the UHSBS provides further evidence to suggest that HIV and HSV-2 prevalence are predictive of one another. Prospective studies are required to test the utility of monitoring HSV-2 prevalence in the newly sexually active as a way of assessing network connectivity/risk of HIV spread.

An alternative explanation for the association between HIV and HSV-2 is that HIV enhances the spread of HSV-2 [21, 43]. This explanation does not, however, fit with the available longitudinal evidence which reveals that populations with high HSV-2/HIV prevalence had equally high HSV-2 prevalences prior to the arrival of HIV [41, 44, 45]. Further studies such as modeling studies would be useful to better delineate the aspects of sexual behavior that are responsible for elevated STI prevalence in certain populations. The results of these studies could then form the basis for interventions targeting these risky behaviors.
